# Who Actually Funds Cancer Research?

**DOI:** 10.1093/jncics/pkz070

**Published:** 2019-10-07

**Authors:** James R Woodgett

**Affiliations:** Lunenfeld Tanenbaum Research Institute, Mount Sinai Hospital, Toronto, Ontario, Canada

Few would argue that support of cancer research is a noble effort that not only has led to significant advances in prevention and treatment over the last 60 years but also has much more to do to control the disease, especially from the Damocles sword of recurrence. The effort is global and comprises a smorgasbord of support mechanisms from small charities to governments and celebrity telethons. In this issue of the Journal, Schmutz et al. (1) survey this plethora of funding sources using a bottom-up approach (involving collection of information from funding source acknowledgments in publications) to assemble the most comprehensive picture yet of the various entities that support cancer research. They arrive at the rather startling number of 4693 organizations that they classify as active funders of cancer research in the past decade. This number represents more than a doubling since 2008, although there are signs of consolidation.

Why is this important? The authors note that it is a starting point from which further analysis can be launched. Its intrinsic worth is not only as an A to Z look-up catalog of a bewildering array of funders but also as a comparator of practice across various jurisdictions. Cancer is unique in disease research in having such a large spectrum of support structures, many of which are well established, starting at the turn of the 20th century. This isn’t the case for other major chronic or infectious diseases with similar morbidities and mortalities such as cardiovascular disease and dementia. The diversity of funders is the consequence of several components including the traditional characterization of the disease by site, the emotional impact of diagnosis, and the survivor and friend communities of the patient population. There was also the perception that not enough was being done fast enough to curb the blight. Some of the most successful efforts coincided with empowerment of women and became a lightning rod for feminist rights and equality. But cancer research spending is far from equitable, with significant disparities between funds raised and incidence and prognosis of the various types of disease. This is due in part to new stigmas such as association of disease with personal lifestyle or by poor survival and, hence, dearth of patient advocates. The atrocious prognosis of lung cancer combines these two elements and has only relatively recently been given the attention warranted by its societal burden.

The study includes supporters of all types of cancer research, from biomedical to health services and behavioral research but did not include interests that focus only on service delivery, advocacy, and so forth. These activities are often part of the mandate and are supported in concert with research, especially by charities. Almost one-third of the listed funding organizations are research facilities, namely hospitals, institutes, and universities. The authors rightly include these in recognition that the full costs of research are rarely covered by external research grants and so on and that the institutions themselves play an indispensable role not only in hosting the research activities but contributing to financial support.

The data reveal some interesting insights. The United States has double the number of cancer research entities than does Europe and almost three times that of Asia. Of course, the gross number doesn’t necessarily reflect the quantity (or quality) of support provided, and this was not assessed in this study. There are some caveats to the methodology (as the authors are careful to explain), including language bias and lack of specificity in identifying the researcher-reported sources of their support. Perhaps surprisingly, more than one-third of publications lacked sufficient information to identify their source of funding —which is increasingly, and understandably, a requirement of funding agencies for purposes of accountability and awareness of the end result of their investment.

It is reasonable to ask why are there so many funders of cancer research, even when institutional contributors are removed. One may also ask if this is the most efficient means to support cancer research, given unavoidable duplication of administrative costs and loss of economy of scale. Taking the data at face value, there is evidence of recent reduction in funder numbers, which may suggest saturation, economic instability, and/or consolidation. The actual causes are important to understand. Regarding the consolidation, prior to the period covered in this study, the largest merger in this sector occurred in 2002 when the Imperial Cancer Research Fund, which was then 100 years old, merged with the Cancer Research Campaign in the United Kingdom. Independently, both charities were highly successful but directly competed for donors, including at their branded main street retail shops that sold donated goods. Prior to the merger to become Cancer Research UK, each had annual incomes of just more than £100 million. In 2017 and 2018, the combined entity had £634 million (2) in revenues, well more than 20 times the income of other United Kingdom–based cancer research charities. Indeed, this is comparable to the largest funder of health research in the United Kingdom, the Medical Research Council, with its total budget for all research being £814 million (3) over the same period (the Wellcome Trust funded £723 million in research in 2018 [4]). It is an excellent example of the power of coherent messages in tackling an enormous challenge.

With the evolution of understanding of tumor etiology, the commonality of certain mutational drivers across subsets of distinct cancer sites, and the critical importance of metastasis as the cause of most cancer deaths, there is an increasing disconnect between cancer tissue site and effective research strategies. Although it is true that the tremendous investment in, for example, breast cancer research, has led to findings that affect other cancers, is there still a place for site-specific research funding agencies? I’d argue there is in the charitable sector, at least, because the motivations to donate are usually emotionally driven and, clinically, patients are likely to continue to be diagnosed by site, even if treated by their mutational landscape. Hopefully, follow-up on studies using this dataset will correlate types of funders with impact of research on outcomes as well as identify gaps in funding and best practices among organizations supporting the drive to control all cancer.

## Note

Affiliation of author: Lunenfeld Tanenbaum Research Institute, Mount Sinai Hospital, Toronto, Ontario, Canada.
